# Dosimetric comparison of three different radiotherapy regimens for bilateral breast cancer

**DOI:** 10.1186/s12938-026-01574-x

**Published:** 2026-05-25

**Authors:** Fanyang Kong, Linyan Xie, Bingyuan Wang, Haiyang Wang, Yawei Li, Meilian Dong, Shujing Shen, Yi Yu

**Affiliations:** 1 Department of Radiation Oncology, The First Affiliated Hospital of Zhengzhou University, Zhengzhou, 45000 China; 2 College of Medical Engineering Xinxiang Medical College, Xinxiang, 453003 China

**Keywords:** Bilateral breast cancer, VMAT, Helical tomotherapy, Dosimetric comparison, LAD

## Abstract

**Objective:**

To compare the dosimetric characteristics of three radiotherapy techniques—VMAT, HT-5, and HT-2.5—in patients with bilateral breast cancer, and to serve as a reference for clinical plan optimization.

**Materials and methods:**

For 30 patients with bilateral breast cancer, radiotherapy plans were generated using Monaco-based volumetric modulated arc therapy (VMAT) and Tomo-based helical tomotherapy with 5 cm field width (HT-5) and 2.5 cm field width (HT-2.5). The prescription dose was 50 Gy in 25 fractions. The conformity index (CI) and homogeneity index (HI) of the planning target volume (PTV) were evaluated. For organs at risk, the *V*_*5*_, *V*_*10*_, *V*_*20*_, *V*_*30*_, and mean dose (*Dmean*) of the lungs and heart, as well as the *Dmean* of the left anterior descending coronary artery (LAD), were analyzed. Beam-on time was recorded for each technique. Statistical analysis and data visualization were performed using R (version 4.3.2).

**Results:**

All three techniques met clinical requirements for target coverage. VMAT achieved the highest CI (P < 0.05), with no significant difference in HI (P > 0.05) among the three techniques. HT-2.5 had a slightly lower Whole Lung-Dmean (10.59 ± 1.09 Gy) than VMAT (10.65 ± 0.69 Gy), but the difference was not statistically significant (P > 0.05). Whole Lung-V5% was significantly lower with HT-5 (52.61 ± 4.90%) (P < 0.05) and HT-2.5 (50.23 ± 4.43%) (P < 0.05) compared to VMAT (57.24 ± 5.37%), while VMAT had significantly lower *V*_*10*_ (P < 0.05)and *V*_*20*_(P < 0.05). For the heart, HT-5 and HT-2.5 provided lower *Dmean* (5.71 ± 1.07 Gy and 5.40 ± 0.94 Gy) (P < 0.05,P < 0.05) and *V*_*5*_ (24.25 ± 8.25% and 21.88 ± 7.37%) (P < 0.05,P < 0.05) than VMAT (6.37 ± 0.95 Gy and 49.52 ± 13.33%). However, heart *V*_*30*_ was significantly lower with VMAT compared to HT-5 (P < 0.05) and HT-2.5 (P < 0.05). LAD *Dmean* was significantly lower with VMAT compared to HT-5 (P < 0.05) and HT-2.5(P < 0.05). Beam-on time was shortest with VMAT (4.5 ± 0.5 min), followed by HT-5 (8.1 ± 0.3 min) and HT-2.5 (14.8 ± 0.6 min).

**Conclusion:**

All three techniques offer acceptable target coverage for bilateral breast cancer. VMAT provides superior target conformity, LAD sparing, and the shortest treatment time. HT-5 and HT-2.5 demonstrate advantages in cardiac protection, with lower heart *Dmean* and *V*_*5*_, while HT-2.5 offers the best reduction in low-dose lung exposure. Considering the combined factors of lung and heart sparing as well as overall treatment time, the Complete block type of 5 cm HT plan can also be recommended for synchronous bilateral breast cancer.

## Introduction

The overall incidence of bilateral primary breast cancer (BPBC) remains relatively low, accounting for approximately 1.4%−12% of all breast cancer cases [1–3]. Synchronous bilateral breast cancer (SBBC) represents 0.4–2.8% of newly diagnosed breast cancers, with a gradually increasing incidence in recent years [4–7]. Radiotherapy for early-stage SBBC poses a considerable challenge due to the complex bilateral thoracic anatomy and proximity to the heart and lungs. while the medial regions are adjacent to critical structures such as the lungs, heart, and liver. This proximity increases the risk of radiation exposure to surrounding organs at risk (OARs), thereby complicating the development of an optimal treatment plan [8–10].

Conventional tangential field techniques, such as three-dimensional conformal radiotherapy (3D-CRT), often result in field overlaps, dose inhomogeneity, and undesirable hot and cold spots[11, 12]. Compared with 3D-CRT, advanced modalities including intensity-modulated radiotherapy (IMRT) [13], volumetric modulated arc therapy (VMAT) [14], and helical tomotherapy (HT) [15] offer improved target dose conformity, enhanced normal tissue sparing, and more favorable cosmetic results. However, the clinical application of HT for SBBC remains relatively underexplored. Variability in treatment planning approaches among dosimetrists has led to inconsistent outcomes, limiting the standardization of HT in this setting and complicating decision-making for radiation oncologists.

**Fig. 1:**
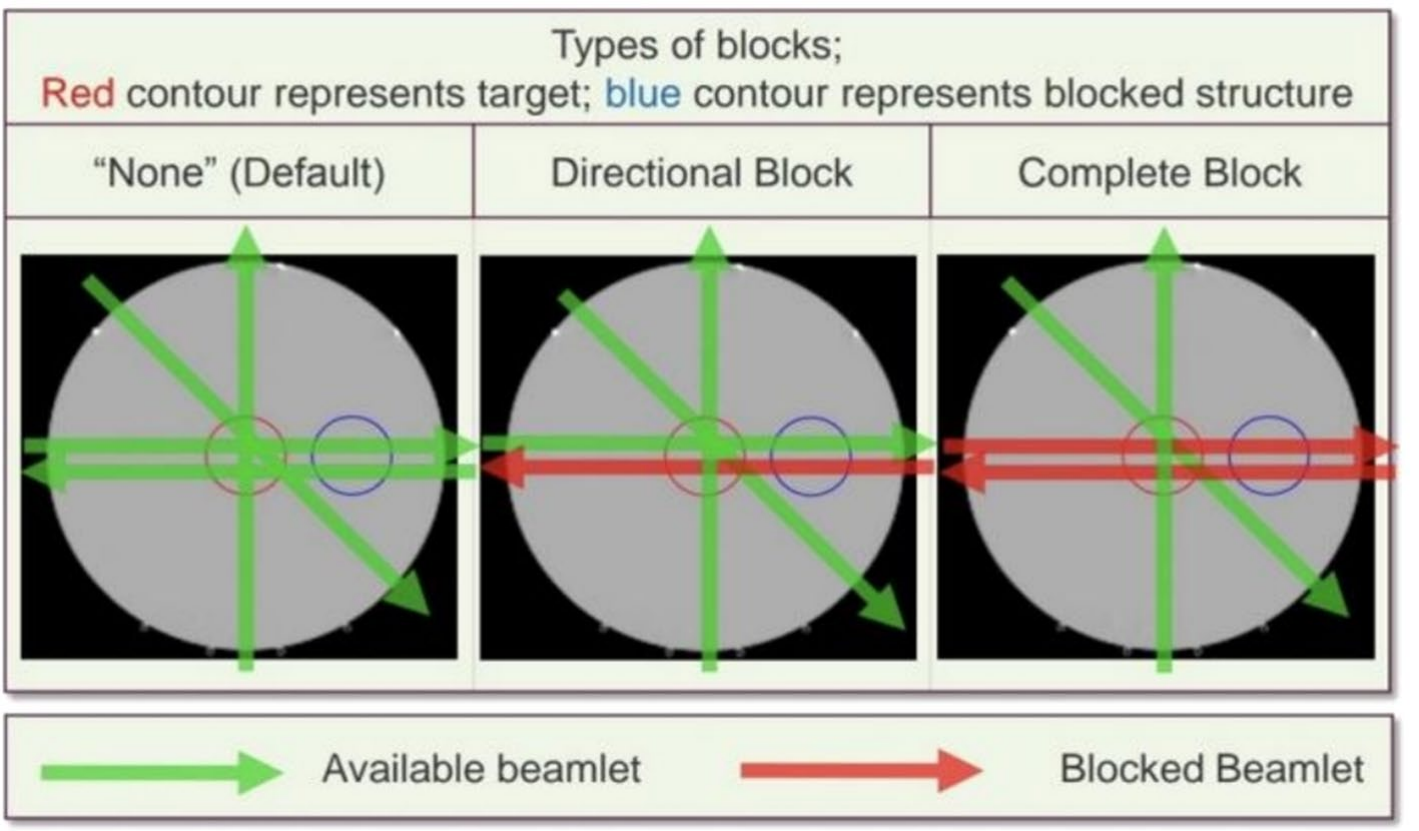
Types of blocks on HT (Blocked beamlets are neglected during all computations)

In this context, our center—which treats approximately 2,000 breast cancer patients annually—conducted a systematic dosimetric comparison of optimized VMAT and HT plans for bilateral breast cancer. VMAT planning was based on Monaco, selecting the optimal protocol from various arc configurations (e.g., single-arc vs. multi-arc, single-isocenter vs. dual-isocenter). For HT, the best plans were selected from different field width and delivery modes (Complete block type vs. Directional block type),as shown in Fig. 1. This study aims to provide a comprehensive dosimetric analysis of VMAT and two HT strategies (HT-5 and HT-2.5), offering practical guidance for selecting optimal radiotherapy techniques for bilateral breast cancer patients.We hypothesized that HT-2.5 offers the greatest low-dose lung sparing compared to HT-5 and VMAT.

## Results

**Fig. 2:**
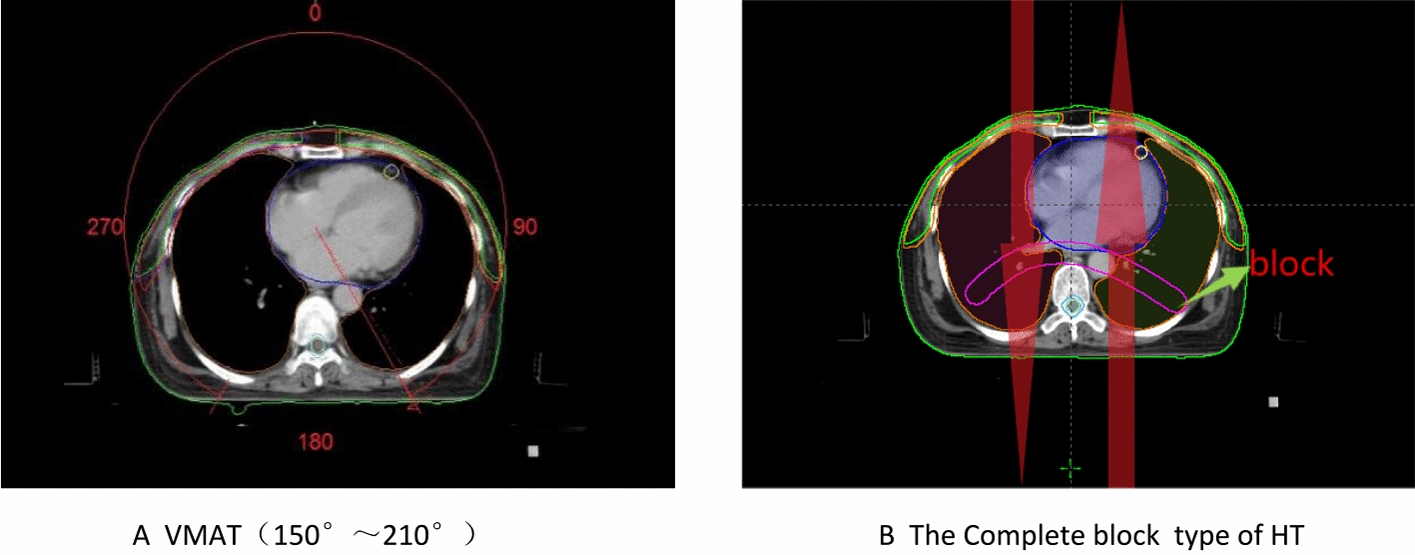
Three bar charts of Whole Lung D_mean_ (Gy), Whole Lung *V*_*5*_ (%), Whole Lung *V*_*10*_ (%) and Whole Lung *V*_*20*_ (%)(n = 30)

**Fig. 3:**
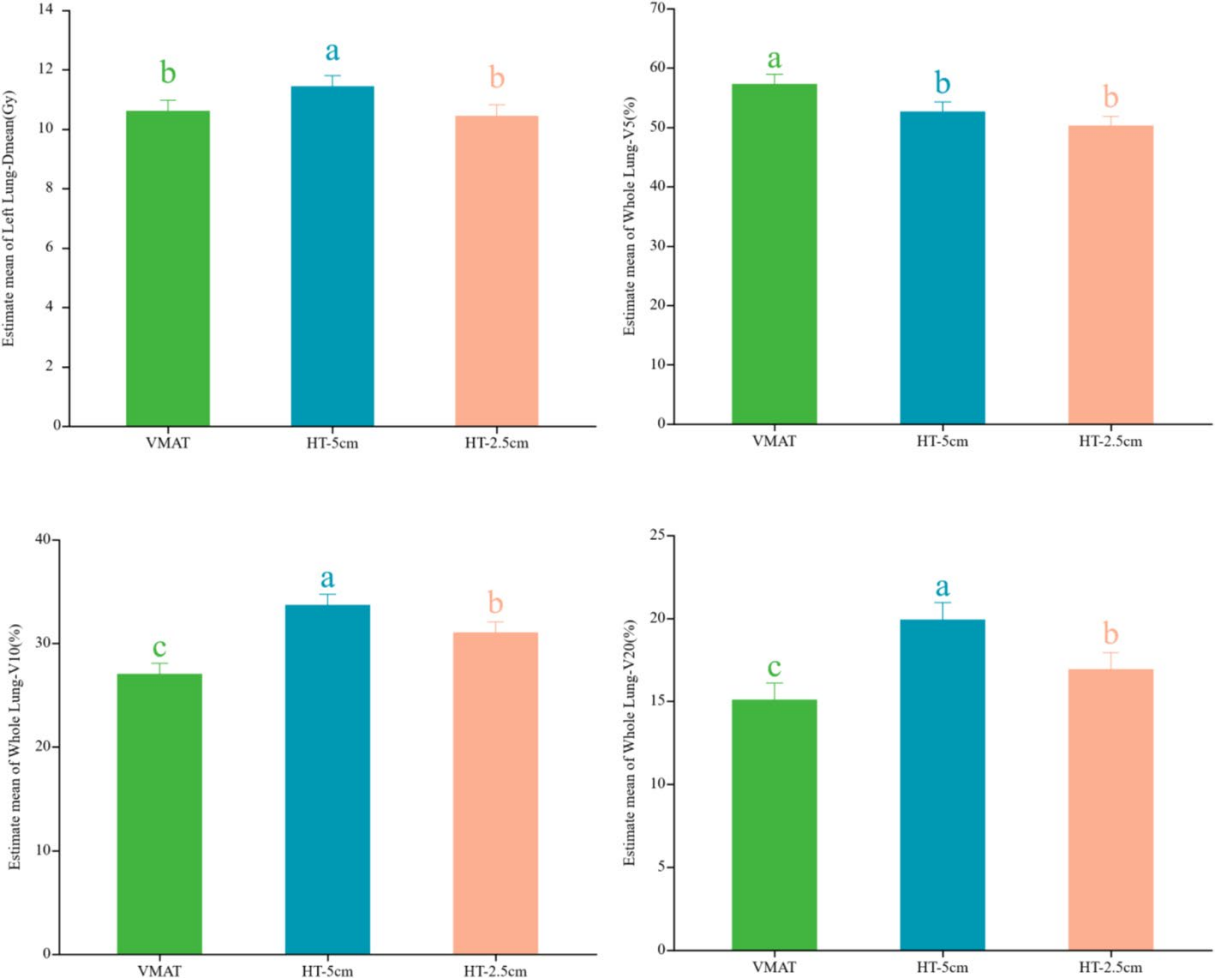
Three bar charts of Heart D_mean_ (Gy), Heart *V*_*5*_ (%), Heart *V*_*10*_ (%) and Heart *V*_*30*_ (%)(n = 30)

**Fig. 4:**
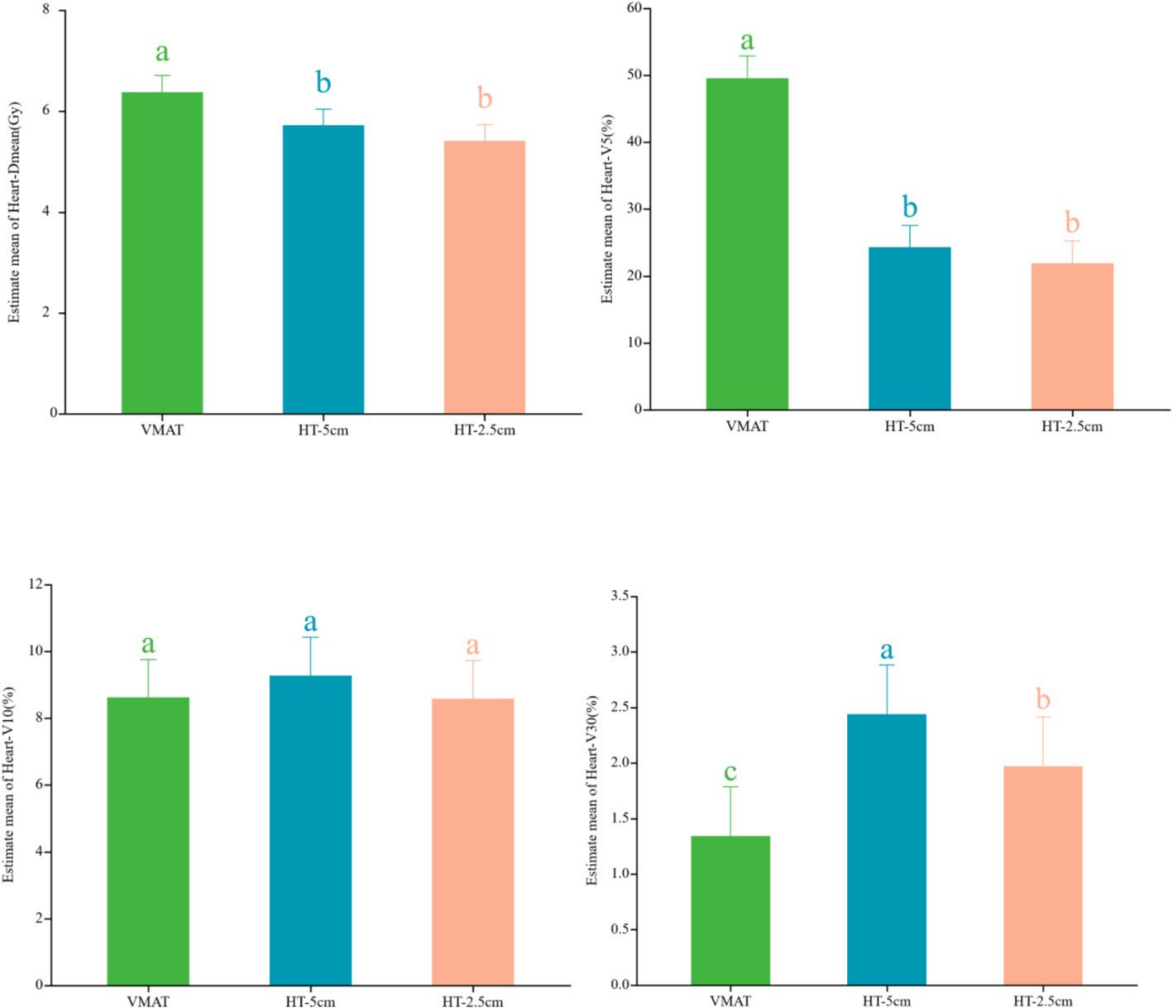
Three bar chart of LAD Dmean(Gy) (n = 30)

**Fig. 5:**
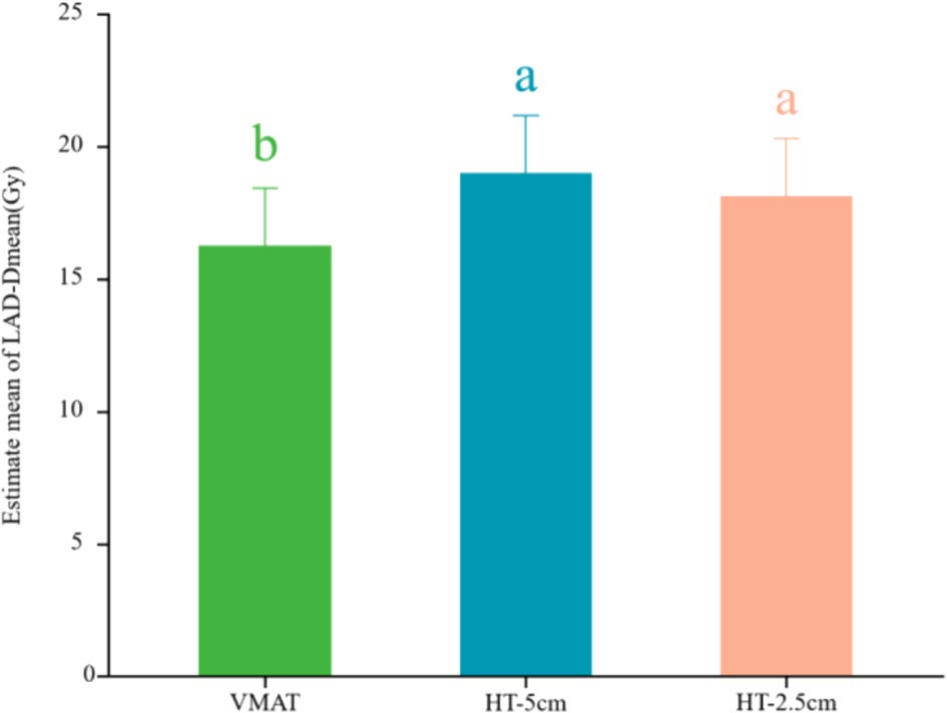
Three bar chart of beam-on time(s) (n = 30)

As shown in Table 1, the *D*_*mean*_, D_95%_, D_98%_, D_2%_, D_50%_, HI and CI of PTV are all expressed. Whether the specific parameters in Figs. 2, 3, 4, 5 are significant also correspond to the P values in Tables 1 and 2. The presence of different letters (a,b, or c) indicates a statistically significant difference (p < 0.05).

**Table 1:** Dosimetric parameters of PTV: mean dose, coverage, conformity index (CI), and homogeneity index (HI)

PTV	G1(VMAT)	G2(HT-5)	G3(HT-2.5)	p value
D_mean_**(Gy)**	52.62 ± 0.39	53.05 ± 0.41	53.07 ± 0.63	−a, −b, −c
D_95%_**(Gy)**	49.92 ± 0.21	50.00 ± 0	50.00 ± 0	−a, −b, −c
D_98%_**(Gy)**	48.10 ± 0.53	47.35 ± 0.47	47.58 ± 0.39	**a, **b,−c
D_2%_**(Gy)**	54.89 ± 0.62	54.49 ± 0.54	54.28 ± 0.67	−a, *b, −c
D_50%_**(Gy)**	52.88 ± 0.44	53.30 ± 0.56	53.50 ± 0.71	-a, *b, −c
HI	0.13 ± 0.01	0.13 ± 0.01	0.13 ± 0.01	−a, −b,−c
CI	0.77 ± 0.03	0.68 ± 0.03	0.69 ± 0.04	**a, **b,-c

a = comparison of G2 vs. G1; b = comparison of G3 vs. G1; c = comparison of G3 vs. G2. *p ≤ 0.05; **p < 0.01; “–a, –b, –c” indicates pairwise comparisons that were not statistically significant (p > 0.05)

**Table 2:** Comparison of organ-at-risk (OAR) dose parameters (lungs, heart, LAD) and beam-on time among VMAT, HT-5, and HT-2.5

Organ	G1 (VMAT)	G2 (HT-5)	G3 (HT-2.5)	p value
Left Lung-D_mean_(Gy)	10.60 ± 1.11	11.43 ± 1.22	10.44 ± 1.09	**a, −b, **c
Left Lung-V_5_(%)	54.93 ± 6.26	52.47 ± 6.00	50.53 ± 6.02	−a, *b, −c
Left Lung-V_10_(%)	26.56 ± 5.21	33.21 ± 4.07	31.13 ± 3.21	**a, **b, −c
Left Lung-V_20_(%)	15.22 ± 3.16	19.48 ± 3.82	16.39 ± 3.02	**a, −b, **c
Left LungV_30_(%)	9.93 ± 2.26	11.68 ± 2.47	9.51 ± 2.27	**a, −b, **c
Right Lung-D_mean_(Gy)	10.71 ± 0.84	11.57 ± 1.34	10.74 ± 1.20	**a, −b, **c
Right Lung-V_5_(%)	58.35 ± 4.75	52.96 ± 5.01	50.21 ± 4.37	**a, **b, −c
Right Lung-V_10_(%)	27.36 ± 2.43	34.01 ± 4.21	31.30 ± 3.42	**a, **b, **c
Right Lung-V_20_(%)	15.01 ± 2.63	19.94 ± 3.76	17.43 ± 3.82	**a, **b, **c
Right Lung-V_30_(%)	9.74 ± 2.25	11.88 ± 2.74	10.03 ± 2.64	**a, −b, **c
Whole Lung-D_mean_(Gy)	10.65 ± 0.69	11.55 ± 1.26	10.59 ± 1.09	**a, −b, **c
Whole Lung-V_5_(%)	57.24 ± 5.37	52.61 ± 4.90	50.23 ± 4.43	*a, **b, −c
Whole Lung-V_10_(%)	27.00 ± 2.75	33.68 ± 3.71	31.02 ± 3.05	**a, **b, *c
Whole Lung-V_20_(%)	15.06 ± 2.25	19.91 ± 3.58	16.90 ± 3.27	**a, **b, **c
Whole Lung-V_30_(%)	9.78 ± 1.93	11.88 ± 2.61	9.76 ± 2.42	**a, −b, **c
Heart-D_mean_(Gy)	6.37 ± 0.95	5.71 ± 1.07	5.40 ± 0.94	**a, **b,−c
Heart-V_5_(%)	49.52 ± 13.33	24.25 ± 8.25	21.88 ± 7.37	**a, **b, −c
Heart-V_10_(%)	8.60 ± 3.82	9.26 ± 3.54	8.57 ± 2.97	−a,−b, −c
Heart-V_20_(%)	3.05 ± 2.07	4.69 ± 2.26	4.21 ± 1.86	**a, **b, −c
Heart-V_30_(%)	1.34 ± 1.29	2.43 ± 1.49	1.97 ± 1.20	**a, **b, *c
LAD-D_mean_ (Gy)	16.25 ± 5.50	18.97 ± 7.17	18.10 ± 6.59	**a, **b, −c
Beam-on time (min)	4.5 ± 0.5	8.1 ± 0.3	14.8 ± 0.6	**a, **b, **c

a = comparison of G2 vs. G1; b = comparison of G3 vs. G1; c = comparison of G3 vs. G2. *p ≤ 0.05; **p < 0.01; “–a, –b, –c” indicates pairwise comparisons that were not statistically significant (p > 0.05)

As shown in Table 1, the prescription dose of 50 Gy for PTV was normalized to 95%. There was no statistical significance among the three groups for the *Dmean*, D_95%_, and HI of the PTV(P > 0.05).VMAT demonstrated the highest CI, which was statistically superior to both HT plans (P < 0.05).

As shown in Table 2, the *Dmean*, *V*_*5*_, *V*_*10*_, *V*_*20*_, and *V*_*30*_ of the Left Lung, Right Lung, Whole Lung, and Heart were expressed, and the average dose *Dmean* of LAD and the beam-on time of the three modes were also included in the table.

As shown in Fig. 2,for the Whole Lung, Dmean was similar between VMAT and HT-2.5, with no significant difference (P > 0.05). HT-5 and HT-2.5 achieved lower *V*_*5*_ values than VMAT (P < 0.05). In contrast, VMAT provided the lowest *V*_*10*_ and *V*_*20*_, which were significantly lower than those of both HT plans (P < 0.05).

As shown in Fig. 3,for the Heart, HT-5 and HT-2.5 reduced Dmean and V_5_ compared to VMAT, with statistically significant differences (P < 0.05). No significant differences were observed among the three groups for V_10_ (P > 0.05). In contrast, VMAT achieved the lowest V_30_, which was significantly lower than both HT plans (P < 0.05).

As shown in Fig. 4, for LAD, VMAT (16.25 ± 5.50 Gy) had the lowest average dose exposure, and this difference was statistically significant compared with HT-5 (18.97 ± 7.17 Gy)(P < 0.05) and HT-2.5 (18.10 ± 6.59 Gy)(P < 0.05).

As presented in Fig. 5, regarding the beam-on time, VMAT has the shortest beam-on time of 4.5 ± 0.5 min. The beam-on time of HT-5 is 8.1 ± 0.3 min, and that of HT-2.5 is 14.8 ± 0.6 min.

## Discussion

Consistent with our hypothesis, HT-2.5 demonstrated the greatest low-dose lung sparing among the three techniques, with a mean *V*_*5*_ of 50.23% compared to 57.24% for VMAT (p < 0.05).

Fiorentino et al. [16] treated 16 patients with synchronous bilateral breast cancer (SBBC) using VMAT, with a prescription dose of 50 Gy in 25 fractions. The mean heart dose was (8.3 ± 3.3) Gy, and no radiation-induced cardiac toxicity was observed at the 2-year follow-up (P < 0.05). Radiation-induced heart injury remains a significant concern in postoperative radiotherapy for breast cancer, and is closely associated with heart *V*_*30*_ and mean heart dose (Dmean) [17]. In our study, the mean heart doses for VMAT, HT-5, and HT-2.5 were 6.37 ± 0.95 Gy, 5.71 ± 1.07 Gy, and 5.40 ± 0.94 Gy.All heart *V*_*30*_ well below the 5% clinical constraint.All three techniques maintained heart doses within the acceptable clinical limits, with HT-5 and HT-2.5 providing superior cardiac sparing.

The lower heart doses observed in our study compared to Fiorentino et al. may be attributed to differences in treatment planning systems and beam delivery optimization. Our VMAT planning utilized Monaco with optimized arc configurations (single-isocenter, three-arc approach with specific angular ranges), whereas variations in planning parameters—even within the same irradiation technique can significantly affect dosimetric outcomes.

All three modalities met clinical constraints for lung dose. However, the low-dose region (particularly *V*_*5*_) is a critical predictor of radiation pneumonitis [18]. In our analysis, the Whole Lung-D_mean_ for VMAT was 10.65 ± 0.69 Gy, with a *V*_*5*_ of 57.24 ± 5.37% and *V*_*20*_ of 15.06 ± 2.25%. For HT-5 and HT-2.5, the Whole Lung-D_mean_ were 11.55 ± 1.26 Gy and 10.59 ± 1.09 Gy, respectively; The Whole Lung-V_5%_ were 52.61 ± 4.90% and 50.23 ± 4.43%, and *V*_*20*_ values were 19.91 ± 3.58% and 16.90 ± 3.27%. Although the HT techniques had a clear advantage in reducing the Whole Lung-V_5%_, their *V*_*10*_ and *V*_*20*_ were significantly higher than those of VMAT(P < 0.05). These findings are consistent with the results reported by Phurailatpam et al. [19], who found that HT offered better control over low-dose spillage but with relatively increased intermediate dose volumes.Statistically significant differences do not always translate into clinically relevant effects and should be interpreted in the context of absolute dose levels and clinical thresholds.

Radiation exposure to the left anterior descending coronary artery (LAD) is a known risk factor for coronary artery disease (CAD) in breast cancer survivors [20, 21]. Thus, minimizing LAD dose is a key objective in treatment planning. In this study, the mean LAD dose was 16.25 ± 5.50 Gy for VMAT, 18.97 ± 7.17 Gy for HT-5, and 18.10 ± 6.59 Gy for HT-2.5, indicating that VMAT provided better LAD sparing.

RCA was not systematically delineated in our study. Although a cardiac atlas includes RCA subsegments, contouring these small vessels on non-contrast planning CT shows substantial inter-observer variability [22]. Clinical studies have reported low RCA doses and weak associations with coronary events [23]. In contrast, the LAD remains the most studied substructure with well-established dose–response evidence, while RCA-specific data are still limited [24].

Regarding treatment delivery time, VMAT had the shortest beam-on time at approximately 4.5 ± 0.5 min, compared to 8.1 ± 0.3 min for HT-5 and 14.8 ± 0.6 min for HT-2.5. Cheng et al. [25] reported a beam-on time of approximately 11.0 min for Tomotherapy using a 2.5 cm field width in SBBC patients, and a mean LAD dose of 19.41 Gy—findings comparable to ours.

A dosimetric study comparing IMRT, VMAT, HT, and intensity-modulated proton therapy (IMPT) in 11 SBBC patients concluded that IMPT offered the most favorable balance between target coverage and organ-at-risk (OAR) sparing, particularly for cardiac structures [26]. Techniques such as deep inspiration breath hold (DIBH) have also demonstrated improved PTV coverage and reduced high-dose OAR exposure when used in conjunction with VMAT [27]. Moreover, flattening filter free (FFF) beams have been shown to reduce treatment time and improve lung protection in SBBC radiotherapy compared with conventional flattened (FF) beams [28, 29]. Lancellotta et al. [30] compared the OAR dose and homogeneity index of HT and TD techniques in SBBC. Compared with the TD technique, HT provided better target coverage and OAR protection.

## Conclusion

All three techniques provide acceptable clinical requirements for bilateral breast cancer. VMAT offers better target conformity, shorter treatment time, and superior protection for the lungs and LAD. HT, especially HT-2.5, provides better cardiac sparing and lower low-dose exposure (*V*_*5*_) to the lungs and heart. Considering the combined factors of lung and heart sparing as well as overall treatment time,the Complete block type of 5 cm HT plan can also be recommended for synchronous bilateral breast cancer.

## Materials and methods

### Inclusion of patients

A total of 30 patients with synchronous bilateral breast cancer who underwent surgery from March 2022 to March 2025 were selected. Among them, 27 cases underwent modified radical mastectomy and 3 cases underwent breast-conserving surgery. The age ranged from 32 to 68 years (median age 45 years).Tumor characteristics were documented for both breasts, including pathological stage, maximum tumor diameter, and histological subtype.Inclusion criteria were: (1) pathologically confirmed SBBC; (2) Karnofsky Performance Status (KPS) ≥ 70; (3) complete wound healing and adequate upper-limb mobility before radiotherapy; and (4) written informed consent obtained, with approval by the institutional ethics committee.Exclusion criteria were: (1) severe cardiac or pulmonary dysfunction; (2) prior irradiation to the chest wall or supraclavicular area; and (3) pregnancy.

### CT positioning

The patient is placed in the supine position and fixed with a carbon fiber breast bracket and thermoplastic film. Metal marking points are placed at the intersection of the positioning laser marking lines for position repetition during treatment, and metal lines are placed at surgical scars and breast tissue. During positioning, a CT scan was performed with a slice thickness of 5 mm, and the scanning range ranged from the mastoid process to the subdiaphragm.No DIBH was applied.CT data was transferred to Monaco and Tomotherapy TPS.

### Target volume delineation

After CT scanning, the data were transferred to the Varian TPS planning system for target volume and organ-at-risk delineation. According to the International Commission on Radiation Units and Measurements (ICRU) Reports 50 and 62, CTV, PTV, and organs at risk were delineated. For breast-conserving surgery: the clinical target volume (CTV) included the bilateral subcutaneous 3 mm breast gland and the underlying chest wall adjacent to the breast gland. For modified radical mastectomy: the CTV included the ipsilateral chest wall, supraclavicular lymph node drainage area, and axillary lymph node drainage area. The planning target volume (PTV) was 5 mm expansion of the CTV and was limited to 3 mm subcutaneously. The bilateral lungs, heart, spinal cord, and left anterior descending coronary artery (LAD) were the main organs at risk.

### Planning design

**Fig. 6:**
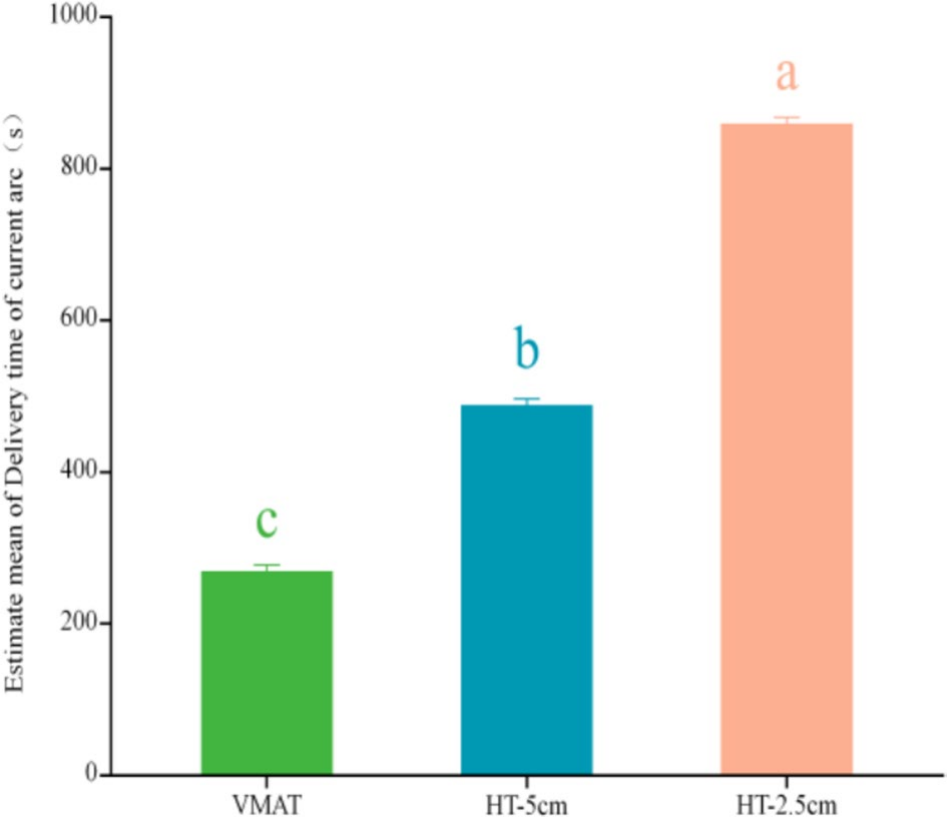
**A** represents the VMAT (a single center, an arc range of 150° to 210°including 3 Arcs); **B** Complete Block type of HT (Red arrows indicate that the beam cannot pass through the block either before or after entering the PTV)

Three types of plans were designed for 30 patients with bilateral breast cancer, including VMAT based on Monaco and HT-5 and HT-2.5 based on HT, as shown in Fig. 6. The three dosimetrists selected all have over 10 years of experience, minimizing the differences in results caused by individual proficiency. VMAT was set with a single center, an arc range of 150° to 210°, and the Max Number of Arcs was 3.The arc ranges were 150° → 210°, 210° → 150°, and 150° → 210°, respectively. The collimator was set at 5°, and the increment was 23°.The dose grid resolution is 3 mm.The Max of Control Points Per Arc was 240.Grid Spacing was 3 mm.The Algorithm is Monte Carlo Photon.The statistical Uncertainty(%) is 0.5.

HT-5 represents a 5 cm field width for HT, and HT-2.5 represents a 2.5 cm field width for HT. G1 represents VMAT, G2 represents HT-5, and G3 represents HT-2.5. The pitch of the HT plan parameters was set at 0.287, the modulation factor (MF) was 3, and the dose calculation grid was set to the Fine mode.

The prescription dose was 50 Gy in 25 fractions. At least 95% of the volume meets the prescribed dose. All plans were independently reviewed by the same radiation oncologist to ensure clinical relevance.The dose limits for OARs were as follows: for both lungs, *V*_*5*_ ≤ 60%, *V*_*10*_ ≤ 30–40%, *V*_*20*_ ≤ 20%, *V*_*30*_ ≤ 10% (Vx represents the volume percentage receiving x Gy dose), *Dmean* ≤ 20 Gy; for the heart, *V*_*5*_ ≤ 30–40%, *V*_*10*_ ≤ 20–30%, *V*_*20*_ ≤ 10–15%, *V*_*30*_ ≤ 5%, *Dmean* ≤ 10–15 Gy; for the liver, *V*_*20*_ ≤ 20%, *V*_*30*_ ≤ 10%, *Dmean* ≤ 15 Gy; for the spinal cord, *Dmax* < 40 Gy; for the thyroid, *V*_*50*_ ≤ 10%; for the humeral head, *V*_*30*_ ≤ 5%; for the LAD, *Dmean* ≤ 10 Gy.

### Radiotherapy plan evaluation

Following the recommendations of ICRU Report 83 [10], the maximum dose D_2%_, minimum dose D_98%_, target coverage D_95%_, median dose D_50%_, mean dose *D*_*mean*_, conformity index CI, and homogeneity index HI of the PTV were evaluated. D_x%_ represents the lowest dose received by x% of the target volume. The formula for calculating CI is: CI = (V_t.ref_/V_t_) x (V_t.ref_/V_ref_). In this formula, V_t.ref_ is the target volume enclosed by the reference isodose surface, V_ref_ is the volume enclosed by the reference isodose surface, and Vt is the target volume. A CI value closer to 1 indicates a higher conformity of the target area. The homogeneity index is defined as HI = (D_2%_ − D_98%_)/D_50%_, and a value closer to 0 indicates better homogeneity of the target area. Evaluation indicators for organs at risk (OAR): *V*_*5*_, *V*_*10*_, *V*_*20*_, *V*_*30*_, and *Dmean* of the left lung, right lung, total lung, and heart; *Dmean* of the left anterior descending artery (LAD). Additionally, the treatment beam-on time of the three plans was statistically analyzed and evaluated.

### Statistical methods

All statistical analyses and chart plotting were conducted in R language (version 4.3.2), with bar charts drawn using the ggplot2 package. A two-sided P < 0.05 was considered statistically significant. The significant difference letter marking method was used to mark the differences between groups.

For between-group analyses, linear mixed-effects models were performed using the R language nlme package. The model employed restricted maximum likelihood (REML) estimation, incorporating treatment groups and individual effects as random effects for correction.

## Data Availability

No datasets were generated or analysed during the current study.
